# Isolation and Characterization of Antimicrobial Compounds in Plant Extracts against Multidrug-Resistant *Acinetobacter baumannii*

**DOI:** 10.1371/journal.pone.0061594

**Published:** 2013-04-22

**Authors:** Yoko Miyasaki, John D. Rabenstein, Joshua Rhea, Marie-Laure Crouch, Ulla M. Mocek, Patricia Emmett Kittell, Margie A. Morgan, Wesley Stephen Nichols, M. M. Van Benschoten, William David Hardy, George Y. Liu

**Affiliations:** 1 Department of Medicine, Cedars-Sinai Medical Center, Los Angeles, California, United States of America; 2 Bothell Research Center, Albany Molecular Research Inc., Bothell, Washington, United States of America; 3 Department of Patient Financial Services, Cedars-Sinai Medical Center, Los Angeles, California, United States of America; 4 Department of Pathology and Laboratory Medicine, Cedars-Sinai Medical Center, Los Angeles, California, United States of America; 5 M. M. Van Benschoten, O.M.D., M.A., C.A. and Associates, Canoga Park, California, United States of America; 6 Department of Pediatrics, Cedars-Sinai Medical Center, Los Angeles, California, United States of America; Aligarh Muslim University, India

## Abstract

The number of fully active antibiotic options that treat nosocomial infections due to multidrug-resistant *Acinetobacter baumannii* (*A. baumannii*) is extremely limited. *Magnolia officinalis*, *Mahonia bealei*, *Rabdosia rubescens*, *Rosa rugosa*, *Rubus chingii*, *Scutellaria baicalensis*, and *Terminalia chebula* plant extracts were previously shown to have growth inhibitory activity against a multidrug-resistant clinical strain of *A. baumannii*. In this study, the compounds responsible for their antimicrobial activity were identified by fractionating each plant extract using high performance liquid chromatography, and determining the antimicrobial activity of each fraction against *A. baumannii*. The chemical structures of the fractions inhibiting >40% of the bacterial growth were elucidated by liquid chromatography/mass spectrometry analysis and nuclear magnetic resonance spectroscopy. The six most active compounds were identified as: ellagic acid in *Rosa rugosa*; norwogonin in *Scutellaria baicalensis*; and chebulagic acid, chebulinic acid, corilagin, and terchebulin in *Terminalia chebula*. The most potent compound was identified as norwogonin with a minimum inhibitory concentration of 128 µg/mL, and minimum bactericidal concentration of 256 µg/mL against clinically relevant strains of *A. baumannii*. Combination studies of norwogonin with ten anti-Gram negative bacterial agents demonstrated that norwogonin did not enhance the antimicrobial activity of the synthetic antibiotics chosen for this study. In conclusion, of all identified antimicrobial compounds, norwogonin was the most potent against multidrug-resistant *A. baumannii* strains. Further studies are warranted to ascertain the prophylactic and therapeutic potential of norwogonin for infections due to multidrug-resistant *A. baumannii*.

## Introduction

Outbreaks of infections due to *Acinetobacter baumannii* (*A. baumannii*) have been reported worldwide [1], and have been attributed to contamination of inanimate objects in the hospital setting and facilitated by healthcare workers who may transmit this organism via direct person-to-person contact [2], [3]. Today, fully active antibiotic options available to treat nosocomial infections due to multidrug-resistant (MDR) *A. baumannii* are extremely limited [1].

Chemotherapeutic agents against MDR *A. baumannii* currently in the pharmaceutical pipeline do not appear to hold promise [3]. In order to identify novel treatment options, commercially available plant extracts were previously screened for their ability to inhibit MDR *A. baumannii in vitro*. The extracts showing the most potent inhibitory effects against a clinical strain of MDR *A. baumannii in vitro* were: *Magnolia officinalis*, *Mahonia bealei*, *Rabdosia rubescens*, *Rosa rugosa*, *Rubus chingii*, *Scutellaria baicalensis*, and *Terminalia chebula* [4].

Tannins, flavones, and phenolic compounds are reported to have low to moderate inhibitory effects on *A. baumannii in vitro* [5], [6]. Tannins are a group of polymerized phenolic substances shown to inhibit a variety of microorganisms [7], [8]. Flavones are phenolic structures that are synthesized by some plants in response to microbial infections [7]. We hypothesized that the anti-MDR *A. baumannii* activity of these plant extracts may result from the combination of tannins and non-tannins that may be present in the extracts. Here, we identified active chemical compounds in the anti-MDR *A. baumannii* plant extracts and characterized their antimicrobial properties *in vitro*.

## Materials and Methods

### Bacterial strains and ethics statement

MDR *A. baumannii* strains 31P, 125P and 152P were isolated from blood (31P) and respiratory (125P and 152P) cultures of three different patients at Cedars-Sinai Medical Center in Los Angeles, California, USA. The strains belonged to different clones based on repetitive-polymerase chain reaction amplification, and their dendrogram is shown in Figure S1 [9]. 31P was determined to be resistant to piperacillin/tazobactam, anti-pseudomonal cephalosporins (ceftazidime and cefepime), carbapenems (imipenem and meropenem), aminoglycosides (tobramycin and amikacin), and fluoroquinolones (ciprofloxacin and levofloxacin) by VITEK®2 (bioMérieux, Durham, North Carolina, USA); and sensitive to colistin by Etest (bioMérieux) based on interpretations according to Clinical and Laboratory Standards Institute (CLSI) breakpoints [10]. The strain was intermediate to tigecycline by Etest with results interpreted per the United States Food and Drug Administration's breakpoint recommendations for *Enterobacteriaceae*. 125P was intermediate to piperacillin/tazobactam, resistant to anti-pseudomonal cephalosporins, carbapenems, fluoroquinolones, colistin and tigecycline, and sensitive to aminoglycosides. 152P was resistant to piperacillin/tazobactam, anti-pseudomonal cephalosporins, aminoglycosides, fluoroquinolones and colistin, and intermediate to carbapenems and tigecycline. We obtained an exempt status from the Cedars-Sinai Institutional Review Board to use these strains to perform all experiments in this study (Protocol number 15767).

*A. baumannii* strain, BAA-1605 and *Escherichia coli* strain, 25922 (*E. coli* 25922) were obtained from the American Type Culture Collection (ATCC, Manassas, Virginia, USA).

### Plant extracts

Seven plant extracts with the most potent inhibitory activity against 31P were selected for further characterization [4]. Dry powders of the plant extracts were obtained as follows: *Magnolia officinalis*, *Rubus chingii*, S*cutellaria baicalensis* and *Terminalia chebula* were obtained from Sun Ten Laboratories, Inc. (Irvine, California, USA); those of *Mahonia bealei* and *Rosa rugosa* were obtained from Bio Essence Corporation (Richmond, California, USA); and those of *Rabdosia rubescens* were obtained from Mayway Corporation (Torrance, California, USA). The minimum inhibitory concentrations (MICs) of these extracts against 31P were identical to those previously reported [4].

### Dimethyl sulfoxide (DMSO) tolerance test

DMSO (Sigma-Aldrich, St. Louis, Missouri, USA) was sterilized using a 0.2 µm Acrodisc nylon membrane syringe filter (Pall, Ann Arbor, Michigan, USA). A 5 mL culture of 31P was grown in cation-adjusted Mueller-Hinton (CAMH) broth (Beckton-Dickenson, Franklin Lakes, New Jersey, USA) with agitation at 37°C. The culture was diluted to 10^6^ CFU/mL in fresh medium. A 100 µL aliquot of CAMH broth with DMSO (concentration range: 0% to 15%) and 100 µL of 31P suspension were mixed in each well of a sterile 96-well polystyrene assay plate (Corning, Lowell, Massachusetts, USA). Negative controls consisted of non-inoculated media. A colistin (Sigma-Aldrich) dose response (0.0625–8 µg/mL) was included as a positive control. Assay plates were incubated without agitation for 16 h at 37°C, and optical density at 600 nm (OD_600 mm_) was measured. Percent growth inhibition (% growth inhibition) for each replicate (n = 8) was calculated as follows: [1−([OD_600 nm_ of a sample – average OD_600 nm_ of negative controls]/[average OD_600 nm_ of positive controls - average OD_600 nm_ of negative controls])]×100. Results were presented as the mean and standard deviation of eight replicates at each DMSO concentration.

### De-tanninization of the plant extracts

1,000 mg of each plant extract powder was solubilized in a solution of 75°C water and DMSO (3∶1 [vol/vol]) at a concentration of 20 mg/mL. Each solution was stirred for 30 min and was centrifuged for 15 min at 7,500 rpm to remove insoluble polysaccharide excipients. The solution was dried down using a Genevac sample concentrator (Genevac Inc, Gardiner, New York, USA) under reduced pressure at 30°C. The sample was re-suspended at a concentration of 10 mg/mL in water and methanol (1∶1 [vol/vol]). 300 mg of polyvinyl pyrrolidone (Crescent Chemical Company, Islandia, New York, USA) was added. The solution was stirred for 30 min and was centrifuged. The supernatant was removed and dried down as described above to yield 150–350 mg of de-tanninized plant extracts.

### Dose response testing of plant extracts before and after de-tanninization

The plant extracts were two-fold serially diluted in water, and 20 µL of solubilized extract and 80 µL of CAMH broth were mixed in an untreated, sterile 96-well plate. This was mixed with 100 µL of a 10^6^ CFU/mL suspension of 31P from a cryopreserved stock. The final concentration of the plant extracts ranged from 7.8125 to 1,000 µg/mL. Negative and positive controls were prepared as described above. Assay plates were incubated without agitation at 37°C, and OD_600 mm_ was measured at 16 h and 24 h. The % growth inhibition was calculated as described above. The same procedure was repeated using the de-tanninized plant extracts. All the samples, crude and de-tanninized, were tested in triplicate.

### Fractionation of de-tanninized plant extracts

Each de-tanninized plant extract was re-suspended at a concentration of 10 mg/mL in water and DMSO (2∶1 [vol/vol]), and a 1 mg aliquot was fractionated using a liquid chromatography/mass spectrometer (LC/MS) system with an ultraviolet (UV), evaporative light scattering detector (ELSD) and MS detectors. ^1^H and ^13^C nuclear magnetic resonance (NMR), correlation spectroscopy (COSY), heteronuclear single-quantum correlation spectroscopy (HSQC) and heteronuclear multiple-bond correlation spectroscopy (HMBC) spectra were recorded using a Bruker DRX 500 NMR spectrometer (Bruker Corporation, Billerica, Massachusetts, USA) in DMSO-d_6_ at 320K at 500 MHz for ^1^H and 125 MHz for ^13^C NMR, respectively. MS was performed on a Sciex API 150 EX single quadrupole (AB SCIEX, Framingham, Massachusetts, USA) with an ion spray ionization source operating in positive mode; capillary voltage, 5.0 kV; declustering potential 35.0. High resolution mass spectra were gathered with a Waters Premier Q-Tof mass spectrometer (Waters, Milford, Massachusetts, USA) equipped with an electrospray ionization source operated in the positive-ion mode; capillary voltage, 3.5 kV; source temperature, 80°C; desolvation temperature, 200°C; nitrogen desolvation flow, 200 l/h. Samples were diluted with water: acetonitrile (1∶1 [vol/vol]) containing 0.1% formic acid and introduced via infusion using the onboard syringe pump. Semi-preparative high performance liquid chromatography (HPLC) was performed using a Waters system (Waters) with a 600 pump connected to a 996 diode-array detector and controlled by Empower software (Empower Software Solutions, Inc., Orlando, Florida, USA).

Each plant extract was fractionated as follows: chromatographic separation was performed at room temperature on a C_18_ Luna 5 µm (100 mm×4.6 mm, inside diameter [i.d.]) column (Phenomenex, Torrance, California, USA). The mobile phase was initially composed of water with trifluoroacetic acid (0.05%) (Solvent A)/acetonitrile with trifluoroacetic acid (0.05%) (Solvent B), 95∶5. The compounds were eluted using an isocratic hold (95∶5, A∶B) until 5 min and then were ramped to 50∶50 from 5 to 15 min, after which a final isocratic step of 50∶50 from 15 to 25 min was used as a wash.

### Identification of active antibacterial compounds

The fractions of all de-tanninized extracts except *Scutellaria baicalensis* were solubilized in 20 µL warm water mixed with 80 µL CAMH broth. The fractions of de-tanninized *Scutellaria baicalensis* were solubilized in 10 µL DMSO, and 2 µL of each fraction was transferred to 98 µL of CAMH broth in a 96-well plate. All wells were mixed with 100 µL of a 10^6^ CFU/mL suspension of 31P. Controls were the same as described above. The assay plates were incubated without agitation for 16 h at 37°C, and OD_600 nm_ was measured. The % growth inhibition was calculated as described above. The experiments were done in triplicate.

The chemical structures of the fractions resulting in >40% of the bacterial growth inhibition were identified on the basis of MS and UV data. *Rosa rugosa*, *Scutellaria baicalensis* and *Terminalia chebula* extracts contained fractions showing >40% growth inhibition. Therefore, 200 mg each of these extracts was prepared at a concentration of 10 mg/mL in water and DMSO (2∶1 [vol/vol]), and chromatographic separation was performed at room temperature on a C_18_ Luna 5 µm (250 mm×10 mm, i.d.) column (Phenomenex). The mobile phase was initially composed of 95∶5 water with trifluoroacetic acid (0.05%) (Solvent A)/acetonitrile with trifluoroacetic acid (0.05%) (Solvent B) and then was ramped to 40∶60 (A∶B) over 30 min. The flow rate was set at 5 mL/min. A complete set of ^1^H NMR, ^13^C NMR, and high resolution mass spectrometry (HRMS) (+electrospray ionization [ESI] time-of-flight mass spectrometry [TOFMS] [M+H]) data were acquired for the peaks of interest. UV spectra, molecular weight and NMR data were used to search internal and external databases (*Dictionary of Natural Products*, Chapman & Hall/CRC Chemical Database, Version 16∶2, Boca Raton, Florida, USA) to identify precise chemical structures of the target compounds.

The following procedures were conducted to isolate more material of the target metabolites from extracts of *Rosa rugosa*, *Scutellaria baicalensis* and *Terminalia chebula*. Preparative HPLC chromatographic separation was performed at room temperature on a C_18_ Prodigy 5 µm (250 mm×21 mm, i.d.) column (Phenomenex). The mobile phase was initially composed of water with trifluoroacetic acid (0.05%) (Solvent A)/acetonitrile with trifluoroacetic acid (0.05%) (Solvent B), 95∶5. The compounds were eluted using an isocratic hold (95∶5, A∶B) until 4 min and then were ramped to 50∶50 from 5 to 25 min, after which a final isocratic step of 50∶50 from 25 to 30 min was used as a wash. The flow rate was set at 20 mL/min. Plant fractions from several HPLC runs were combined and collected into vials, and dried down using a Genevac sample concentrator under reduced pressure at 30°C. The purity of the isolated fractions was confirmed by HPLC and/or ^1^H NMR.

### Determination of the minimum inhibitory concentration (MIC)_90_, and minimum bactericidal concentration (MBC), time-kill kinetic analysis, and resazurin reduction assay

The MIC_90_ was defined as the lowest concentration of a compound which inhibited ≥90% of bacterial growth compared to an untreated control. Purified forms of the following compounds were solubilized at 10 mg/mL in warm water: ellagic acid from *Rosa rugosa*; chebulagic acid, chebulinic acid, corilagin and terchebulin from *Terminalia chebula*. The solutions were two-fold serially diluted in water, and 20 µL per well was transferred to 80 µL CAMH broth in a 96-well plate. Each solution was mixed with 100 µL of either 31P or BAA-1605 suspension (5×10^5^ CFU/mL final). Assay plates including negative control (CAMH broth only) and positive control (bacterial suspension at a final concentration of 5×10^5^ CFU/mL) were incubated without agitation for 16 h at 37°C, and OD_600 nm_ was measured. The % growth inhibition was calculated as described above. Purified norwogonin was insoluble in water and was solubilized at 25.6 mg/mL in DMSO and two-fold serially diluted. One µL of norwogonin dilution was transferred to 99 µL of CAMH broth in a 96-well plate, mixed, and 20 µL per well were transferred to 4 wells of a 384-well plate. Negative controls (CAMH broth plus 1% DMSO) and positive controls (same as above) were included. Norwogonin, having the lowest MIC_90_, was tested against BAA-1605 in the same fashion.

Next, MBC testing and time-kill kinetic assays of norwogonin were performed on 31P. The bactericidal effect was defined as a 99.9% decrease in CFU (3 logs) in the starting inoculum during a 24 h incubation in the presence of antibiotic. The MBC was determined by transferring 1 µL from each well of an overnight MIC plate to 63 µL of sterile CAMH broth in a fresh 384-well plate. OD_600 nm_ was measured after 20 h incubation at 37°C. The % growth inhibition was calculated as described above. For both the MIC and MBC assays, 12 replicate wells were tested. For the time-kill kinetic analysis, a bacterial overnight culture was diluted (5×10^5^ CFU/mL final) using CAMH broth supplemented with DMSO (1% final) and 1× or 2× MIC of norwogonin. Cultures were grown with agitation at 37°C, and aliquots were collected at the indicated time intervals, serially diluted in 0.9% sterile saline solution and plated onto CAMH agar plates. Viable colonies were enumerated after 24 h at 37°C. The limit of detection for this preliminary assay was 10^1^ CFU/mL.

Finally, the growth inhibitory effect of norwogonin against 31P, 125P and 152P was determined by measuring both turbidity and respiration. Purified norwogonin was solubilized at 12.8 mg/mL in DMSO and triplicate two-fold serial dilutions (0.003 mg/mL final concentration) were performed. Five hundred nL of each dilution was transferred to three 384-well assay plates. Each assay plate was inoculated with 50 µL of 31P, 125P, or 152P diluted to 5×10^5^ CFU/mL in CAMH broth, and incubated without agitation for 16 h at 37°C. Negative controls (CAMH broth plus 1% DMSO) and positive controls (inoculum plus 1% DMSO) were included in each assay plate. Turbidity was assessed by reading OD_600 nm_. After determining turbidity, 5 µL of a 0.001% aqueous resazurin solution (Sigma-Aldrich) was added to each well and assay plates were incubated at room temperature for 30 min. Resazurin reduction to resorfurin was determined by measuring fluorescence (530 nm excitation/590 nm emission). These assays were performed in triplicate on three separate days.

### Dose response testing of norwogonin in combination with synthetic anti-Gram negative bacterial agents

Stock solutions of ampicillin, cefepime, sulbactam, sulfamethoxazole (Fisher Scientific, Pittsburgh, Pennsylvania, USA), azithromycin, levofloxacin, minocycline, rifampin and trimethoprim (Sigma-Aldrich) were prepared at 12.8 mg/mL in DMSO. Stock solutions of colistin (Sigma-Aldrich) and tobramycin (Fisher) were dissolved in water. Imipenem (USP, Rockville, Maryland, USA) solution was warmed to 50°C for 5 min to facilitate solubilization, aliquoted, and stored at −20°C. Ampicillin and sulbactam were combined in a ratio of 2∶1 while trimethoprim and sulfamethoxazole were combined in a ratio of 5∶1. The same ratios are used for commercially available co-formulated ampicillin/sulbactam (2∶1) and trimethoprim and sulfamethoxazole (5∶1). The MIC_90_ values of antibiotics against *E. coli* 25922 were determined in triplicate experiments on two separate days, and were consistent with CLSI performance standards [10].

MIC_90_ of eight synthetic antibiotics and two synthetic antibiotic combinations against 31P, BAA-1605 and *E. coli* 25922 were determined using a modified broth micro-dilution methods as described by CLSI [11]. 20 µL of bacterial culture diluted to 10^6^ CFU/mL in CAMH broth were dispensed to 384-well plates containing 20 µL of two-fold serial dilutions of the antibiotics in CAMH broth. The antibiotics were tested in the following 12-point two-fold serial dilutions series: 0.016–64 µg/mL for ampicillin/sulbactam, azithromycin, colistin, imipenem, levofloxacin, minocycline and rifampin; 0.031–128 µg/mL for trimethoprim/sulfamethoxazole; and 0.063–256 µg/mL for cefepime and tobramycin. Final DMSO concentration in the assay was 1%. Positive and negative controls were included as described above. Plates were incubated without agitation for 16 h at 37°C, and OD_600 nm_ was measured. The % growth inhibition was calculated as described above. Combinations of norwogonin and chemotherapeutic agents were tested as follows: half maximal inhibitory concentration (IC_50_) of norwogonin was extrapolated from the graph of bacterial growth inhibition. Testing of norwogonin in combination with each of the above synthetic antibiotics and antibiotic combinations against 31P was performed in the same fashion, except that in addition to the synthetic antibiotic dose response, CAMH broth was supplemented with norwogonin either at its IC_50_ (16 µg/mL) or a concentration one step below the IC_50_ (8 µg/mL). The experiment was performed in duplicate on two separate days. The combination study was repeated with the same strain using each of the above synthetic antibiotics at a concentration that was one step below their respective IC_50_ and a dose response of norwogonin. The experiment was performed in triplicate on one day.

## Results

### Dose response testing of crude and de-tanninized plant extracts

As DMSO was used to solubilize *Scutellaria baicalensis* extracts, norwogonin and many of the synthetic antibiotics, the DMSO tolerance of 31P was evaluated prior to MIC and MBS determination. As shown in Figure S2, growth of 31P decreased with increasing DMSO concentration. The reduction in growth was most pronounced above 2% DMSO in the medium. Therefore, all subsequent assays were performed at or below a 1% DMSO final concentration.

Due to their non-specific protein binding capacity, tannins are known to interfere with the isolation and purification of bioactive compounds [12]; therefore, the antibacterial effect of the various plant extracts was determined before and after de-tanninization. Results of dose response testing of our plant extracts before and after de-tanninization are shown in Figure S3. At concentrations between 7.81 and 1,000 µg/mL, the antimicrobial potency of the four extracts (*Rosa rugosa*, *Rubus chingii*, *Scutellaria baicalensis* and *Terminalia chebula*) was reduced after de-tanninization by 13.5 to 39.3% (Figures S3D–G). In this concentration range, *Magnolia officinalis*, *Mahonia bealei* and *Rabdosia rubescens* showed no inhibitory activity before or after de-tanninization (Figures S3A–C).

### Isolation and characterization of antimicrobial compounds

UV chromatograms of the seven extracts from the LC/MS system are shown in Figure S4. Eighty fractions from each extract were tested against 31P *in vitro*, and the chemical structures of the fractions inhibiting >40% of the bacterial growth were identified on the basis of MS and UV data as follows: ellagic acid, which is a phenolic natural antioxidant in *Rosa rugosa*; norwogonin, which is a flavonoid in *Scutellaria baicalensis*; chebulinic acid, corilagin and terchebulin, all of which are ellagitannins in *Terminalia chebula*; and chebulagic acid, which is a benzopyran tannin antioxidant in *Terminalia chebula*. Chromatograms of *Rosa rugosa*, *Scutellaria baicalensis* and *Terminalia chebula* extracts from preparative HPLC are shown in Figure S5. Peaks with a retention time of 13.783 min corresponded to ellagic acid (Figure S5A); retention time of 16.958 min corresponded to norwogonin (Figure S5B); and retention times of 9.129, 10.946, 12.931 and 14.443 min corresponded to terchebulin, corilagin, chebulagic acid and chebulinic acid, respectively (Figure S5C).

The purity of norwogonin was confirmed to be >95% by HPLC and ^1^H NMR, and that of ellagic acid, chebulagic acid, chebulinic acid, corilagin and terchebulin was confirmed to be >85% by HPLC.

**Figure 1:**
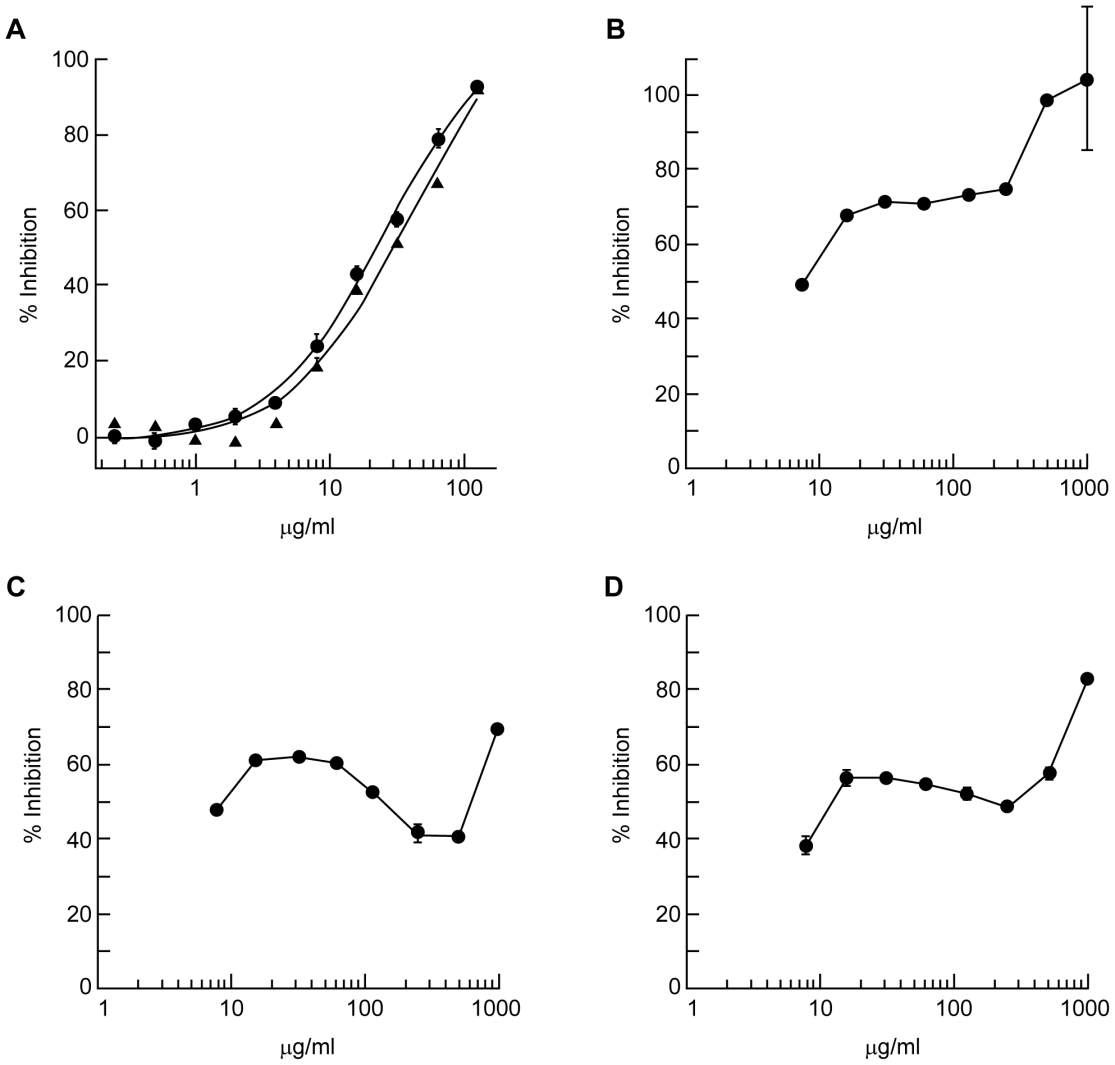
Determination of antimicrobial activity of purified compounds from plant extracts against two *A.* baumannii** strains. Two-fold serially diluted norwogonin (**A**), terchebulin (**B**), chebulagic acid (**C**) and corilagin (**D**) suspensions were prepared in cation-adjusted Mueller-Hinton broth and mixed with an equal volume of either strain 31P or BAA-1605 suspension (5×10^5^ CFU/mL final). Bacterial growth was measured after a 16 h incubation at 37°C. The final test concentration for each compound ranged from 0.25 to 128 µg/mL for norwogonin (MIC_90_ = 128 µg/mL), and 7.8 to 1,000 µg/mL for terchebulin (MIC_90_ = 500 µg/mL), chebulagic acid and corilagin. • 31P and ▴ BAA-1605.

**Figure 2:**
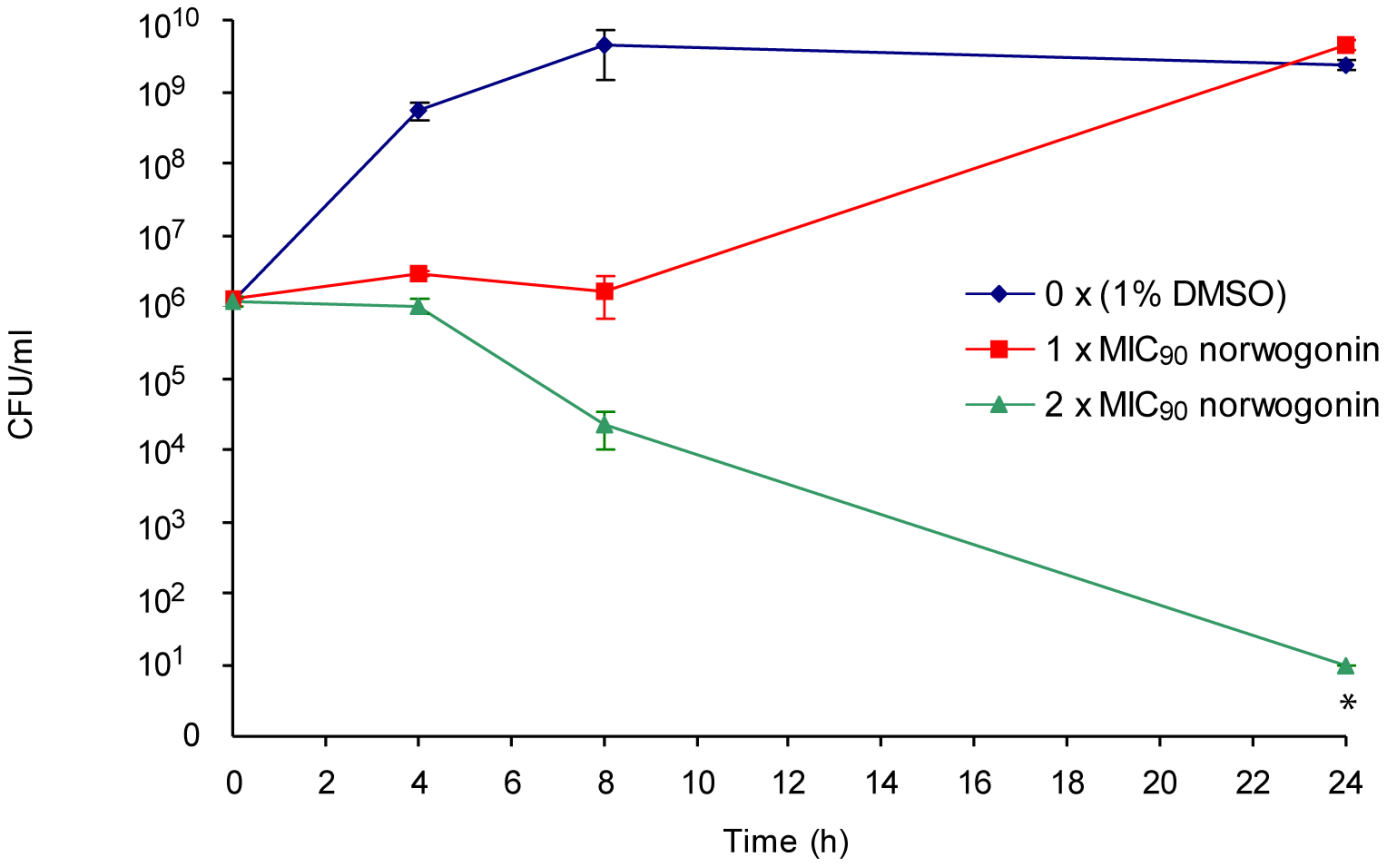
Time-kill kinetic analysis of norwogonin against 31P. The time-kill kinetics of 31P by norwogonin at 1× and 2× MIC was studied over a 24 h incubation. Aliquots were collected at 0, 4, 8 and 24 h, serially diluted in phosphate buffered saline before plating on Mueller-Hinton agar plates. Surviving colonies were enumerated after an 18 h incubation at 37°C. * Estimate: no colonies were observed at the highest concentration plated.

The most potent plant-derived compound was determined to be norwogonin with an MIC_90_ of 128 µg/mL against both 31P and BAA-1605 (Figure 1A). The MIC_90_ of terchebulin, the second most potent compound, was 500 µg/mL (Figure 1B). The chemical formula and structures of norwogonin and terchebulin are shown in Figure S6. Time-kill kinetic analysis of norwogonin against 31P showed complete growth inhibition at 2×MIC (256 µg/mL), and no re-growth was observed at 24 h (Figure 2).

**Figure 3:**
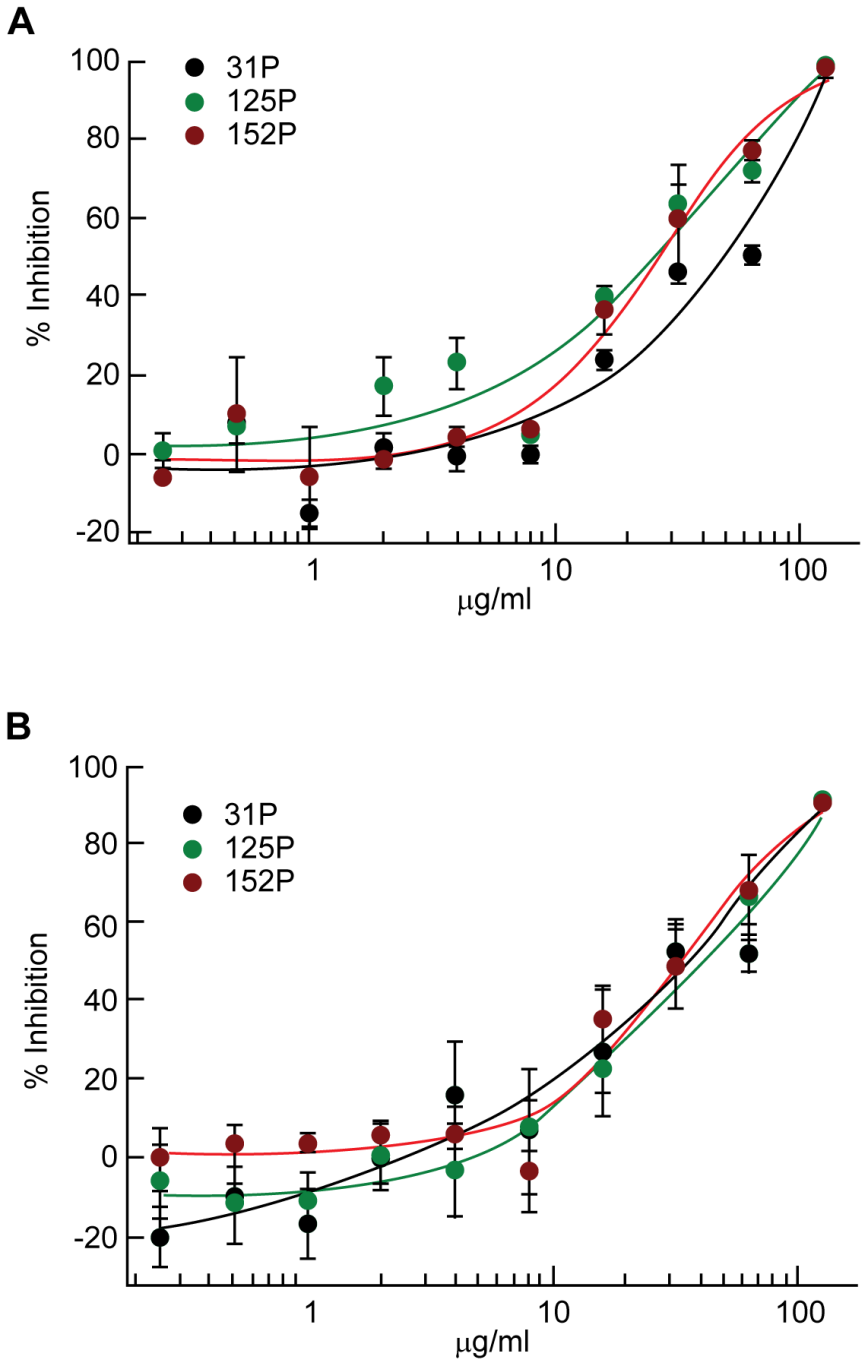
MIC_90_ determination of purified norwogonin against three clonally distinct strains of *A. baumannii*. MIC_90_ of norwogonin against 31P, 125P and 152P was determined by measuring OD_600 nm_ (**A**) and was confirmed by resazurin reduction assay (**B**).

The MIC testing of norwogonin was repeated against 31P, 125P and 152P to confirm that it had the same growth inhibitory effects on *A. baumannii* strains that are clonally distinct and that have different antimicrobial susceptibility profile. In this experiment, MIC_90_ was determined by measuring turbidity. A resazurin reduction assay was performed in parallel to confirm MIC_90_ as, in the absence of cell lysis, turbidity cannot distinguish between live and dead bacteria [13]. Resazurin, an oxidation-reduction indicator, has been used to assess bacterial viability and to test for antimicrobial activity [14]–[16]. MIC_90_ of norwogonin against 31P, 125P and 152P was 128 µg/mL in all experiments by turbidity measurement (Figure 3A) and resazurin reduction assay (Figure 3B).

As shown in Figure 1A, dose-response testing of norwogonin demonstrated a sigmoidal growth inhibition curve (Figure 1A). Terchebulin demonstrated a biphasic growth inhibition curve (Figure 1B). Growth inhibition reached 70% at 31.25 µg/mL and remained steady up to 250 µg/mL before increasing to 90% inhibition at concentrations greater than 250 µg/mL. This biphasic growth inhibition curve was also seen in chebulagic acid and corilagin (Figures 1CD).

The following compounds did not reach the 90% inhibition threshold: ellagic acid inhibited 67% at 250 µg/mL; chebulagic acid inhibited 60.39% at 62.5 µg/mL and 88% at 1,000 µg/mL; chebulinic acid inhibited 65% at 62.5 µg/mL; and corilagin inhibited 56% at 15.625 µg/mL and 83% at 1,000 µg/mL.

### Dose response testing of norwogonin in combination with anti-Gram negative bacterial agents

Ten synthetic antibiotics and antibiotic combinations belonging to different classes and clinically relevant for the treatment of a variety of Gram-negative bacterial infections were selected and tested alone or in combination with norwogonin for growth inhibitory activity against 31P.

When tested alone at 8 and 16 µg/mL against 31P, norwogonin resulted in 40% and 53% growth inhibition, respectively. The IC_90_ values for synthetic antibiotic alone or in combinations with either 8 or 16 µg/mL of norwogonin are shown in Table 1. None of the ten synthetic antibiotics or antibiotic combinations displayed significant enhancement in their anti-*A. baumannii* activity when tested in the presence of norwogonin. Similarly, dose response testing of norwogonin in the presence of a fixed dose of synthetic antibiotics did not demonstrate a shift in inhibitory activity (data not shown).

**Table 1:** Dose response testing of synthetic anti-Gram negative bacterial agents in combination with norwogonin.

Anti-Gram negative bacterial agents	IC_90_ of antibacterial agents alone	IC_90_ in the presence of 8 µg/mL norwogonin	IC_90_ in the presence of 16 µg/mL norwogonin
Ampicillin/sulbactam	5.8	4.8	4.9
Azithromycin	2.1	2.0	2.7
Cefepime	31	23	30
Colistin	0.66	1.6	1.2
Imipenem	3.6	2.6	3.9
Levofloxacin	31	27	38
Minocycline	1.0	0.71	0.78
Rifampin	2.0	1.2	2.0
Tobramycin	80	69	89
Trimethoprim/sulfamethoxazole*	-	-	-

The IC_90_ (µg/mL) of each antibiotic either alone or in combination with 8 or 16 µg/mL norwogonin against strain 31P were determined. Results are presented as the average IC_90_ for two experiments each done in duplicate.

* IC_90_ for trimethoprim/sulfamethoxazole could not be determined as maximum inhibition remained below 90% at all concentrations tested.

## Discussion

In this study, the most potent non-tannin fraction was identified as norwogonin (5,6,7-trihydroxyflavone) from *Scutellaria baicalensis*, with an MIC_90_ of 128 µg/mL against clonally distinct clinical strains of *A. baumannii* as well as the ATCC strain BAA-1605. Ellagic acid, chebulinic acid, chebulagic acid, corilagin and terchebulin had low to moderate anti-*A. baumannii* activity *in vitro*.

To our knowledge this is the first report of anti-*A. baumannii* activities for norwogonin although inhibitory activities of *Scutellaria baicalensis* and its constituents, baicalin and baicalein, against other bacteria are documented in the medical literature. For example, baicalin was shown to inhibit *Chlamydia trachomatis* by down-regulating the expression of its serine protease, Chlamydia protease-like activity factor [17], [18]; ethanol extract of *Scutellaria baicalensis* was reported to have mild growth inhibitory effects on *Salmonella enterica* serovars Typhimurium, Kentucky, Senftenberg, and Enteritidis *in vitro* [19]; baicalin and *Scutellaria baicalensis* were found subsequently to be bactericidal against *Helicobacter pylori* based on broth dilution assays [20]; the mechanism of growth inhibition in the latter two studies remains unknown. A study by Chan *et al* (2011) demonstrated that a combination of baicalein and ciprofloxacin synergistically inhibited quinolone-resistant strains of Methicillin-resistant *Staphylococcus aureus in vitro*, and baicalein was shown to inhibit enzymatic activity of staphylococcal pyruvate kinase [21].

Terchebulin, chebulagic acid and corilagin in *Terminalia chebula* demonstrated a two-step killing kinetic. We have previously demonstrated the skip-well phenomenon of *Terminalia chebula* (i.e. an observation of regrowth after a clearly defined point of bacterial inhibition in broth dilution) [4]. However, at this time, it is unclear whether the seemingly biphasic nature of growth inhibition with terchebulin, chebulagic acid and corilagin in *Terminalia chebula*, is related to the previously observed skip-well phenomenon.

In the medical literature, several phenolic compounds from plant extracts are reported to enhance the potency of synthetic antibiotics against *A. baumannii in vitro*. For example, ellagic and tannic acids were reported to enhance the activity of novobiocin, coumermycin, chlorobiocin, rifampicin and fusidic acid against *A. baumannii in vitro* [22]. Synergy was noted between a purified polyphenol in green tea and topical mafenide against a clinical strain of MDR *A. baumannii in vitro* [5]. In contrast, we observed no evidence of additivity or synergy in the activity of combinations of norwogonin and anti-Gram negative antibiotics. In this study, combining norwogonin with ellagic acid, chebulagic acid, chebulinic acid, corilagin or terchebulin did not produce synergy against 31P *in vitro* either (data not shown).

This research project was a critical initial part of our effort to develop new therapeutics and infection-control modalities for MDR *A. baumannii*. Development of norwogonin as a systemic therapeutic may be limited by its high MIC against *A. baumannii*. In drug development where unfavorable factors (e.g. limited bioavailability and high toxicity) limit systemic use of novel compounds, their topical application has been tested to address superficial infections and colonization as exemplified by synthetic antimicrobial peptides [23]. A key to controlling MDR *A. baumannii* outbreaks is identification and elimination of its source [2], [3], [24]. Studies have shown that antibacterial prophylaxis with topical and systemic agents can decrease respiratory tract infections in critically ill patients [25]. Indeed, it is suggested that adjunctive control measures to decolonize MDR *A. baumannii* from patients' skin should be explored [2]. Colistin, which is considered a last resort systemic antibiotic for infections due to MDR *A. baumannii* and is available in a topical form at a 1,000× the systemic concentration, can still result in resistance in *A. baumannii*, as colistin usage increases [1]. It would seem to be more prudent to reserve colistin for the treatment of life-threatening infections due to MDR *A. baumannii*, and to utilize different antimicrobial agents for its decolonization. Therefore, further studies are warranted to ascertain the prophylactic and therapeutic potential of norwogonin for infections due to MDR *A. baumannii*.

## Supporting Information

Figure S1 **Dendrogram of 31P, 125P and 152P.** The strains were analyzed by repetitive-polymerase chain reaction amplification (PCR); PCR products were separated by a gel matrix. Band patterns for each strain were aligned and interpreted as described in our previous study [9].
(TIF)

Figure S2 **Dose response testing of dimethyl sulfoxide (DMSO) against 31P.** Growth of 31P was measured after a 16 h incubation at 37°C in cation-adjusted Mueller-Hinton broth supplemented with increasing concentration of DMSO.
(TIF)

Figure S3 **Dose response testing of crude and de-tanninized extracts against** ***A. baumannii*** **strain 31P.** The ability of crude and de-tanninized extracts of *Magnolia officinalis* (**A**) *Mahonia bealei* (**B**), *Rabdosia rubescens* (**C**), *Rosa rugosa* (**D**), *Rubus chingii* (**E**), *Scutellaria baicalensis* (**F**), and *Terminalia chebula* (**G**) to inhibit growth of 31P was evaluated by measuring optical density at 600 nm (OD_600 nm_) after a 16 h incubation of 5×10^5^ CFU/mL suspension in cation-adjusted Mueller-Hinton broth supplemented with increasing concentration (7.8125–1,000°g/mL) of each extract
(TIF)

Figure S4 **Ultraviolet chromatogram of the seven extracts from the liquid chromatography/mass spectrometry system.** *Magnolia officinalis* (**A**), *Mahonia bealei* (**B**), *Rabdosia rubescens* (**C**), *Rosa rugosa* (**D**), *Rubus chingii* (**E**), *Scutellaria baicalensis* (**F**), and *Terminalia chebula* (**G**).
(TIF)

Figure S5 **Chromatograms of** ***Rosa rugosa*** **,** ***Scutellaria baicalensis*** **and** ***Terminalia chebula*** **extracts from high performance liquid chromatography.** The chemical structures of the fractions that resulted in >40% of the bacterial growth inhibition corresponded to peaks with retention times of 13.783 min from *Rosa rugosa* (**A**); 16.958 min from *Scutellaria baicalensis* (**B**); and 9.129, 10.946, 12.931 and 14.443 min from *Terminalia chebula* (**C**). The precise chemical structures were elucidated by liquid chromatography/mass spectrometry analysis and nuclear magnetic resonance spectroscopy.
(TIF)

Figure S6 **Chemical structure of the most potent compounds in this study.** Norwogonin (**A**) in *Scutellaria baicalensis* (chemical formula C_15_H_10_O_5_), and terchebulin (**B**) in *Terminalia chebula* (chemical formula C_48_H_28_O_30_).
(TIF)
