# Supplementary figures and images for: Isolation and Characterization of Antimicrobial Compounds in Plant Extracts against Multidrug-Resistant Acinetobacter baumannii
Source: PLoS One. 2013 Apr 22;8(4):e61594. doi: 10.1371/journal.pone.0061594 (PMC3632535; doi:10.1371/journal.pone.0061594)

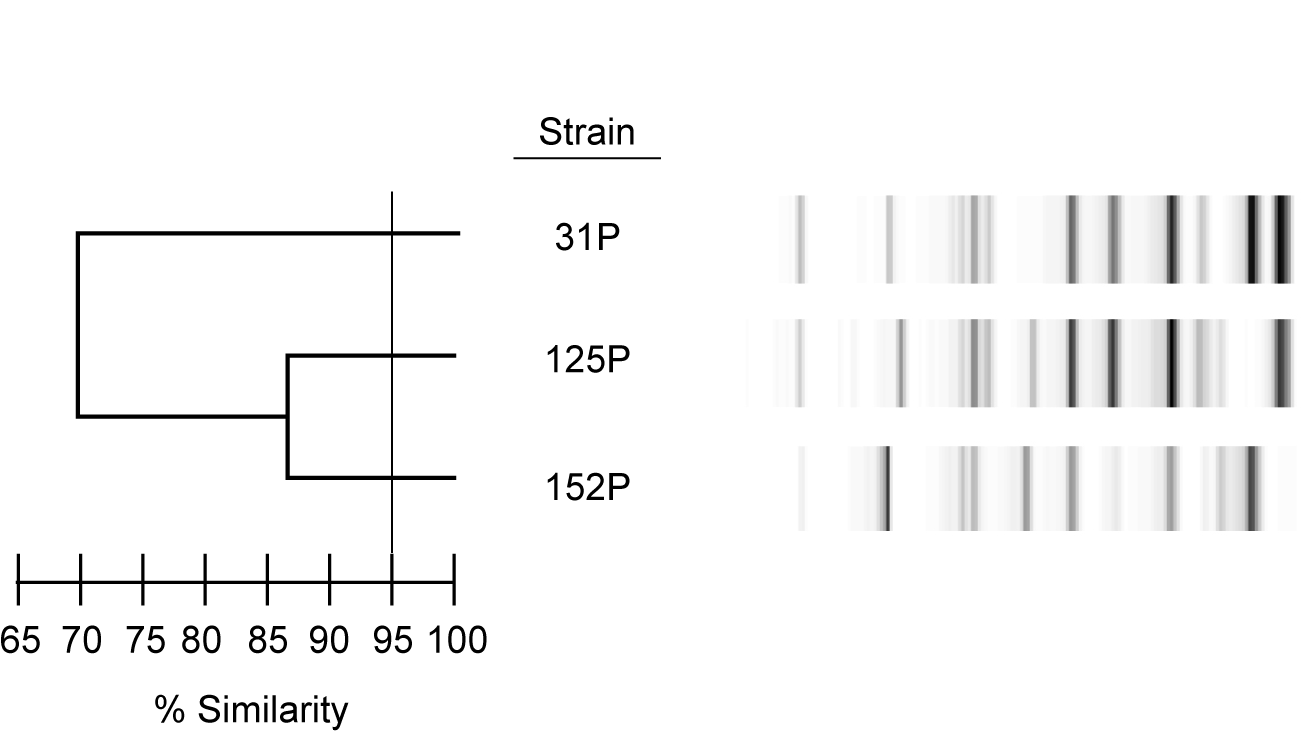

Supplement: Figure S1 — Dendrogram of 31P, 125P and 152P. The strains were analyzed by repetitive-polymerase chain reaction amplification (PCR); PCR products were separated by a gel matrix. Band patterns for each strain were aligned and interpreted as described in our previous study [9]. (TIF) [file pone.0061594.s001.tif]

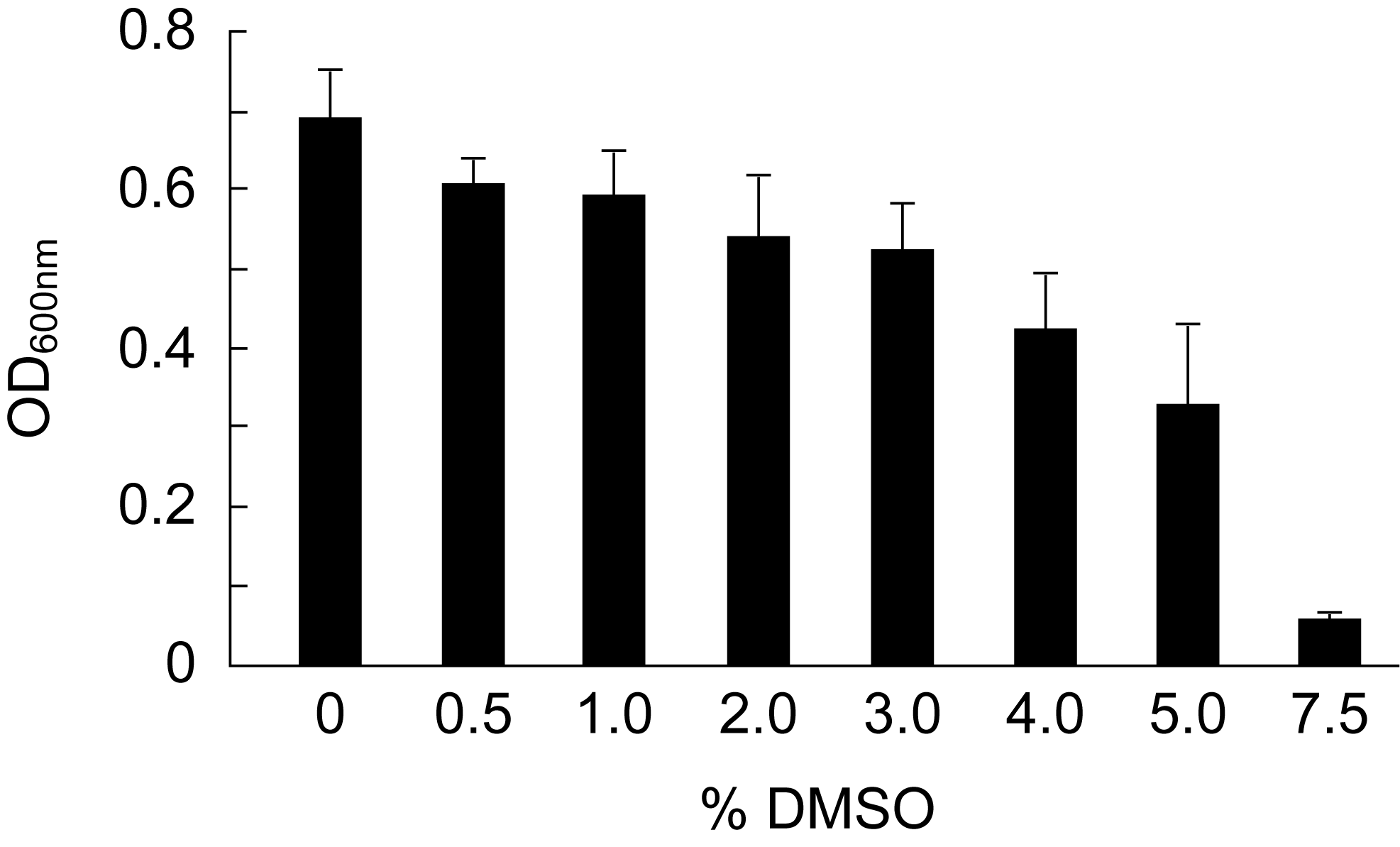

Supplement: Figure S2 — Dose response testing of dimethyl sulfoxide (DMSO) against 31P. Growth of 31P was measured after a 16 h incubation at 37°C in cation-adjusted Mueller-Hinton broth supplemented with increasing concentration of DMSO. (TIF) [file pone.0061594.s002.tif]

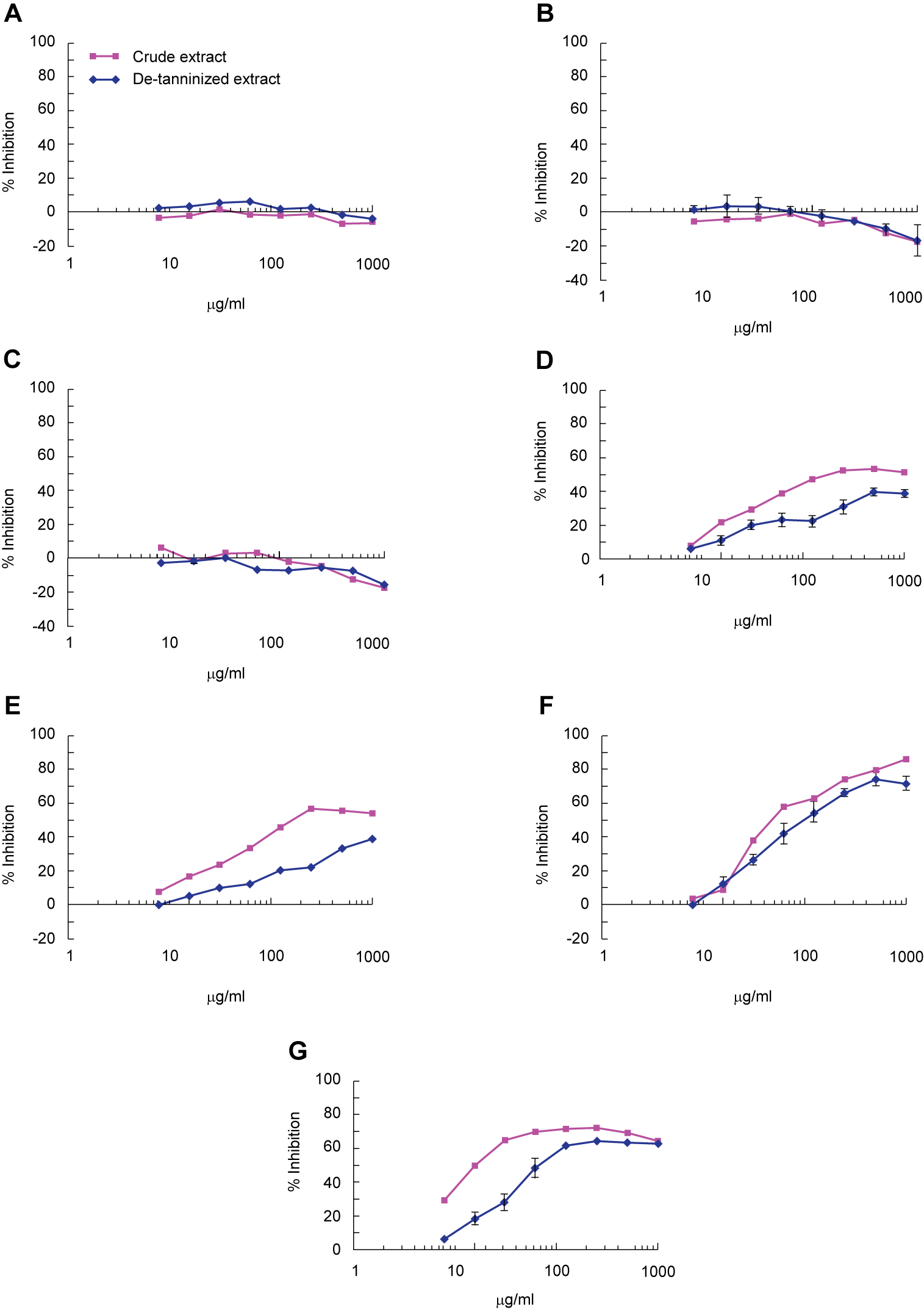

Supplement: Figure S3 — Dose response testing of crude and de-tanninized extracts against A. baumannii strain 31P. The ability of crude and de-tanninized extracts of Magnolia officinalis (A) Mahonia bealei (B), Rabdosia rubescens (C), Rosa rugosa (D), Rubus chingii (E), Scutellaria baicalensis (F), and Terminalia chebula (G) to inhibit growth of 31P was evaluated by measuring optical density at 600 nm (OD600 nm) after a 16 h incubation of 5×10^5^ CFU/mL suspension in cation-adjusted Mueller-Hinton broth supplemented with increasing concentration (7.8125–1,000°g/mL) of each extract (TIF) [file pone.0061594.s003.tif]

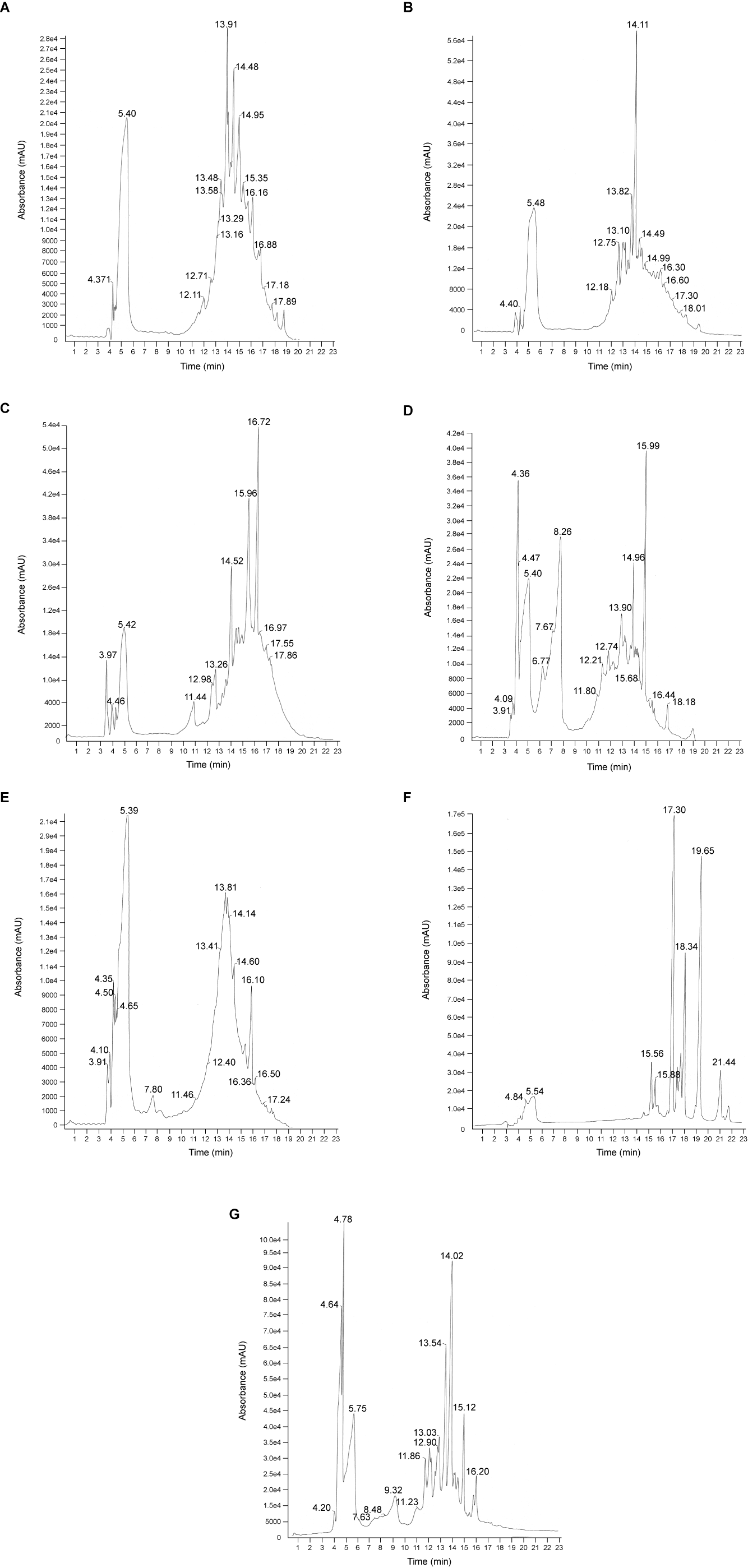

Supplement: Figure S4 — Ultraviolet chromatogram of the seven extracts from the liquid chromatography/mass spectrometry system. Magnolia officinalis (A), Mahonia bealei (B), Rabdosia rubescens (C), Rosa rugosa (D), Rubus chingii (E), Scutellaria baicalensis (F), and Terminalia chebula (G). (TIF) [file pone.0061594.s004.tif]

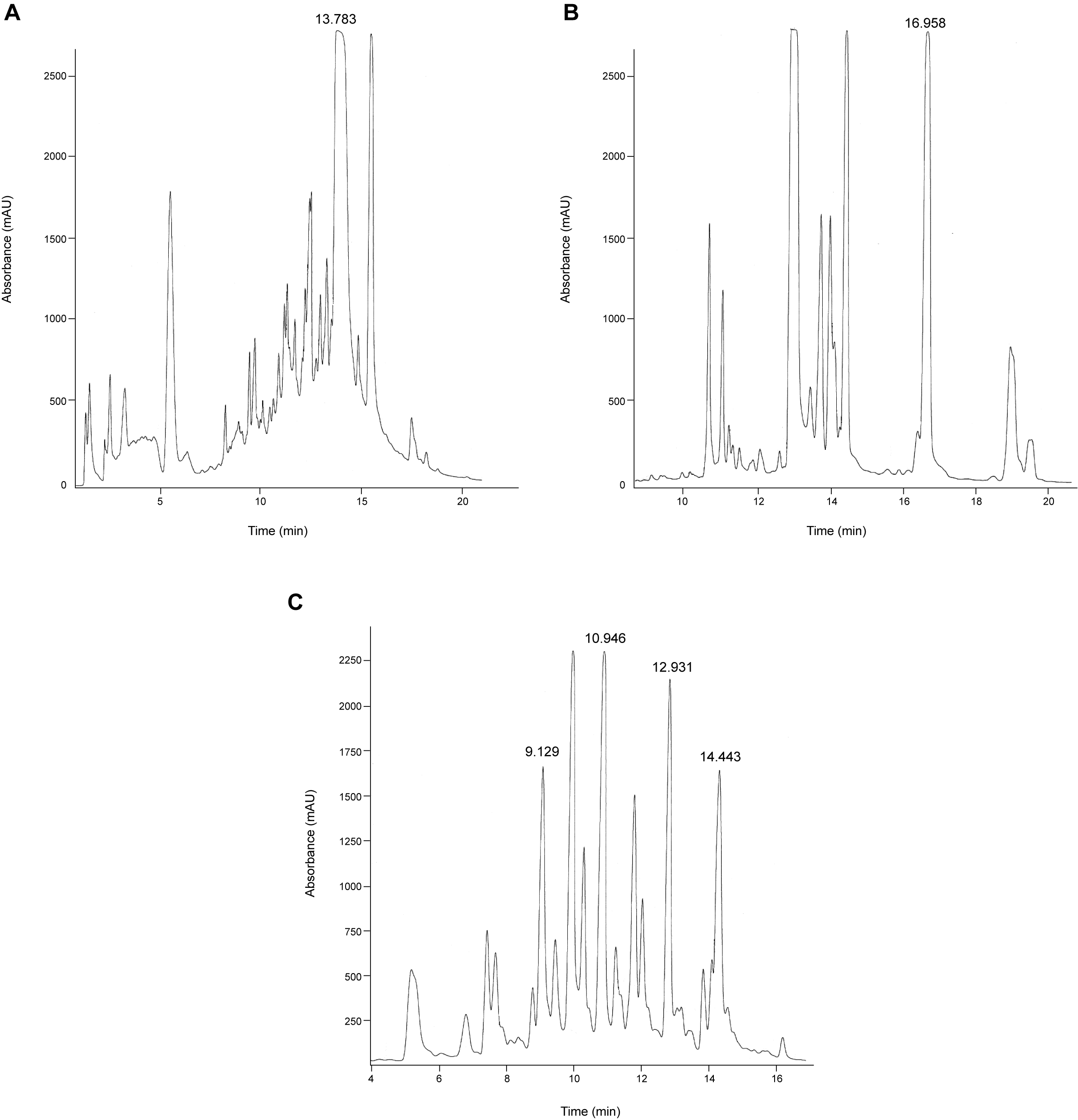

Supplement: Figure S5 — Chromatograms of Rosa rugosa , Scutellaria baicalensis and Terminalia chebula extracts from high performance liquid chromatography. The chemical structures of the fractions that resulted in >40% of the bacterial growth inhibition corresponded to peaks with retention times of 13.783 min from Rosa rugosa (A); 16.958 min from Scutellaria baicalensis (B); and 9.129, 10.946, 12.931 and 14.443 min from Terminalia chebula (C). The precise chemical structures were elucidated by liquid chromatography/mass spectrometry analysis and nuclear magnetic resonance spectroscopy. (TIF) [file pone.0061594.s005.tif]

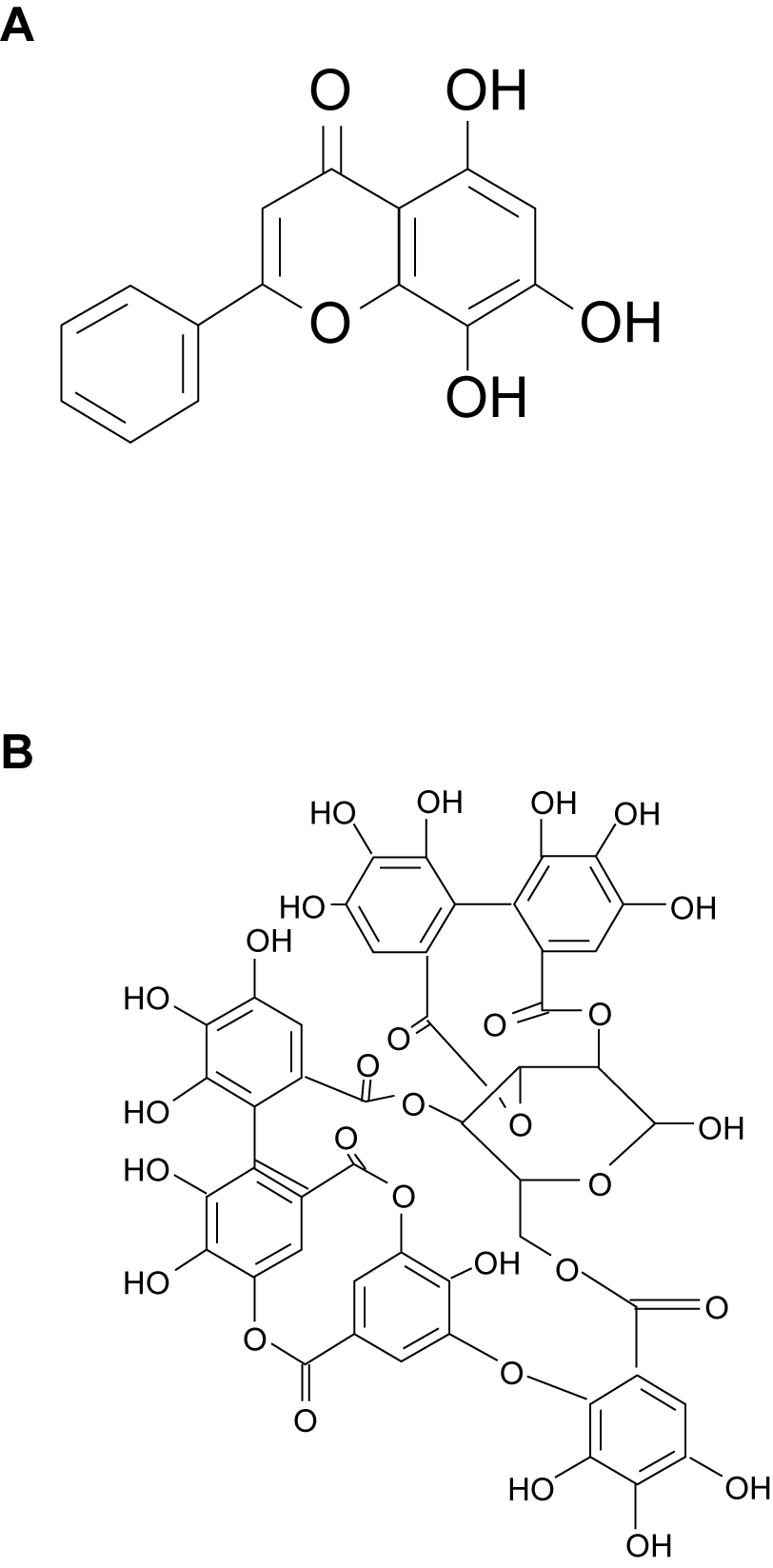

Supplement: Figure S6 — Chemical structure of the most potent compounds in this study. Norwogonin (A) in Scutellaria baicalensis (chemical formula C15H10O5), and terchebulin (B) in Terminalia chebula (chemical formula C48H28O30). (TIF) [file pone.0061594.s006.tif]
